# Prevalence of diabetic macular edema among type II diabetic patients in tele-screening program using OCT and fundus photos in a tertiary hospital, Riyadh, Saudi Arabia

**DOI:** 10.1186/s40942-025-00747-5

**Published:** 2025-11-07

**Authors:** Abdullrahman Mohammed Alshehri, Mohammed Naji Almutairi, Mishari Alqahtani, Abdullmajeed Alfakhri

**Affiliations:** 1 College of Medicine, King Saud Bin Abdulaziz University for Health Sciences, Riyadh, Saudi Arabia; 2 Ophthalmology Division, National Guard Health Affairs, Riyadh, Saudi Arabia

**Keywords:** Diabetic macular edema, Screening, Prevalence

## Abstract

**Purpose:**

To provide local data on the prevalence and risk factors of diabetic macular edema in the Ministry of National Guard Health Affairs, Riyadh, Saudi Arabia.

**Method:**

A cross-sectional cohort study was conducted to screen the prevalence of diabetic macular edema among diabetic patients, with 906 patients randomly selected between May 2023 and September 2023 in a tele-screening program using OCT and fundus photos at the Ministry of National Guard Health Affairs, Riyadh, Saudi Arabia.

**Results:**

In a sample of 906 diabetic patients who participated in a tele-screening program, 38 cases of diabetic macular edema (DME) were diagnosed, resulting in a prevalence of 4.19%. Males and females each composed 50% of the sample. The average age was 61.39 years (SD = 10.114), and 31.6% had diabetes for over 15 years. Most patients had comorbidities, including hypertension (86.8%) and dyslipidemia (89.5%). Diabetic complications were common: neuropathy was present in 23.6%, and nephropathy was present in 21%. Among the 48 eyes assessed, the mean central macular thickness was 315 ± 69 μm. The distribution of diabetic retinopathy severity in 48 eyes included 17 (35%) no retinopathy, 8 (17%) mild NPDR, 15 (31%) moderate NPDR, 7 (15%) severe NPDR, and 1 (2%) PDR. Center-involved DME was found in 45.8% of the eyes, whereas non-center-involved DME was observed in 54.2% of the eyes.

**Conclusion:**

This study in Riyadh, Saudi Arabia, revealed a 4.19% prevalence of diabetic macular edema (DME), emphasizing the importance of regular screening, especially for patients with longstanding diabetes, hypertension, and dyslipidemia.

## Introduction

Diabetes mellitus (DM) is a chronic systemic disease that has increased in prevalence in Saudi Arabia and globally over time [1]. Diabetic retinopathy (DR) is defined as an abnormality in the vasculature of the fundus and is divided into 2 stages on the basis of ophthalmological findings and the level of severity. The initial stage, non-proliferative diabetic retinopathy (NPDR), involves microaneurysms, retinal hemorrhages, hard exudates due to chronic leakage around retinal vessels, and venous dilation, which results in retinal ischemia. The subsequent stage, proliferative diabetic retinopathy (PDR), is identified by the emergence of abnormal new retinal vessels, referred to as neovascularization, which can occur at the optic disk or elsewhere. When fluid accumulation and swelling of the retina occurs, this is called diabetic macular edema (DME), which is found at any stage of DR, but it is more common in more severe stages. Furthermore, DME and PDR are major causes of blindness in DR patients worldwide [2]. In DME, there is disruption of the blood‒retinal barrier, allowing the entry of proteins and other solutes into retinal tissue that are typically confined to the bloodstream, subsequently leading to fluid accumulation and decreased visual acuity. Additionally, edema can progress, leading to irreversible tissue damage, causing the death of retinal cells, and resulting in permanent visual impairment [3]. The screening and diagnosis of DME can be accomplished through methods such as slit lamp bio-microscopy, fundus photography, and optical coherence tomography (OCT). The diagnosis of DR is made based on clinical manifestations of vascular abnormalities in the retina [4].

OCT is a noninvasive imaging technique that uses light waves to take cross-section pictures of the retina. It usually takes 5–10 min, and it is used for monitoring disease progression and for suspected retinopathy. It is an important tool to screen patients with diabetes annually for the development of retinal disease. Based on a recently published meta-analysis, the overall prevalence of DME was 5.47% (95% CI: 3.66%–7.62%). In a breakdown, it was 5.81% (95% CI: 0.07%–18.51%) in low- to middle-income countries and 5.14% (95% CI: 3.44%–7.15%) in high-income countries. This finding indicates that approximately 5.5% of individuals with diabetes have DME, with a statistically nonsignificant lower prevalence observed in high-income countries than in low- to middle-income countries [4]. Moving on to local data, Al-Rubeaan and colleagues collected data via the Saudi national diabetes registry (SNDR) and reported that the overall prevalence of diabetic retinopathy was 9936 patients (19.7%), with 2876 (5.7%) having DME [5]. Another study conducted locally in Al-Madinah AL-Munawarah reported a prevalence of DR of 36.1%, of which 6.4% was proliferative DR in a random sample of 690 diabetic patients [6]. In the Jizan district, 740 participants underwent full diabetic retinopathy screening and ophthalmic evaluation, including a dilated fundus exam [7]. Overall, 206 (27.8%) patients had DR, and 58 (7.8%) had DME. Additionally, the prevalence of DR was 30.5% among diabetic patients in eastern Saudi Arabia (Al-Hasa) [8]. Saudi Arabia is considered one of the countries with the highest prevalence rates of diabetes mellitus (DM) and associated complications, with regional disparities. The occurrence of DME varies from 6% to 10% among individuals with diabetes in Saudi Arabia. However, cases of DME in the country are often underreported in Saudi Arabia, which could impact patient care [9]. Our study aims to provide local data about the prevalence and risk factors for diabetic macular edema detected through a tele-screening program for diabetic retinopathy patients via fundus photography and optical coherence tomography (OCT) at the Ministry of National Guard Health Affairs, Riyadh, Saudi Arabia.

## Methods

After IRB approval was obtained from King Abdullah International Medical Research Center (KAIMRC), a cross-sectional cohort study was conducted to screen the prevalence of diabetic macular edema among diabetic patients in a tele-screening program via OCT and fundus photos. Fundus photographs and optical coherence tomography (OCT) images were obtained using the Topcon DRI OCT Triton Plus (Topcon Corporation, Tokyo, Japan) for all the patients. These screening tools are integrated into the electronic health record platform BestCare through the Axis Server. As the program is connected to the BestCare EMR system, all patient records are centralized.

The study was conducted at the Ministry of National Guard Health Affairs in Riyadh, Saudi Arabia. Fundus photos and OCT were obtained at various primary healthcare clinics to screen for diabetic retinopathy. The study included 906 type 2 diabetic patients who were selected randomly from May 2023 until September 2023 in the tele-screening program. OCT and fundus photos were obtained as part of routine tele-screening visits. Trained technicians conducted the imaging procedures following standardized protocols established by the tele-screening program. Data on patient demographics, diabetes duration, and glycemic control were extracted from electronic medical records. Every patient who was suspected of having DME had a follow-up appointment with an ophthalmologist for a thorough ophthalmic examination.

The primary outcome variable was the prevalence of diabetic macular edema, which was diagnosed on the basis of OCT and fundus photo findings according to established criteria. The covariates included age, sex, duration of diabetes, HbA1c levels, diabetic complications, hypertension, dyslipidemia, other comorbidities, smoking status, GLP-1 receptor agonist status, presence of diabetic retinopathy (1 = no retinopathy, 2 = mild NPDR, 3 = moderate NPDR, 4 = severe NPDR, 5 = PDR), central macular thickness, type of DME, visual acuity, intraocular pressure (IOP), lens status, history of trauma, prior intraocular surgery, and previous intravitreal anti-VEGF treatment.

Diabetic macular edema was defined as the presence of retinal thickening in the macular area on OCT, accompanied by characteristic findings on fundus photography. The classification of DME followed the guidelines set by the International Council of Ophthalmology (ICO) for diabetic eye care [10]. DME was categorized into two groups: center-involved DME and non-center-involved DME. Center-involved DME refers to retinal thickening in the macula that affects the central subfield zone and is typically 1 mm (approximately 0.04 in diameter). On the other hand, non-center-involved DME is characterized by retinal thickening in the macula that does not encompass the central subfield zone.

The data obtained from the study were analyzed via SPSS version 23 software. Descriptive statistics were used to summarize and describe the sample population’s characteristics. The incidence of diabetic macular edema was calculated as the proportion of diabetic patients diagnosed with DME during the study period. Specifically, the number of patients diagnosed with DME was divided by the total number of diabetic patients included in the study. The calculated incidence was subsequently expressed as a percentage. Measures such as the mean, standard deviation, median, and range were calculated for continuous variables such as age, HbA1c levels, and central macular thickness. Frequencies and percentages were computed for categorical variables such as sex, duration of diabetes, presence of associated conditions (hypertension, dyslipidemia), smoking status, GLP-1 receptor agonist use, and diabetic complications (diabetic retinopathy, neuropathy, nephropathy). Excel was utilized to create visually appealing graphs and charts representing various aspects of the data.

## Results

Patient demographic data, including sex, age, HbA1c score, diabetic complications, and comorbidities, were obtained and are shown in Table 1.

**Table 1:** Patients’ demographics

Characteristic	Value (*n* = 38)
Male, n (%)	19 (50%)
Female, n (%)	19 (50%)
Mean age (years), mean ± SD	61.39 ± 10.11
Age group 25–44, n (%)	5 (13.2%)
Age group 45–64, n (%)	18 (47.4%)
Age group ≥ 65, n (%)	15 (39.5%)
Mean HbA1c (%), mean ± SD	9.1 ± 1.7
Peripheral neuropathy, n (%)	16 (42.1%)
Nephropathy, n (%)	9 (23.6%)
Hypertension, n (%)	33 (86.8%)
Dyslipidemia, n (%)	34 (89.5%)
Smoker, n (%)	4 (10.5%)
Received GLP-1 RA, n (%)	3 (7.8%)

This table summarizes the baseline demographic and clinical characteristics of patients (*n* = 38) diagnosed with diabetic macular edema (DME) in the study

Abbreviations: SD Standard Deviation, HbA1c Hemoglobin A1c, GLP-1 RA Glucagon-Like Peptide-1 Receptor Agonist, n Number of patients

**Fig. 1:**
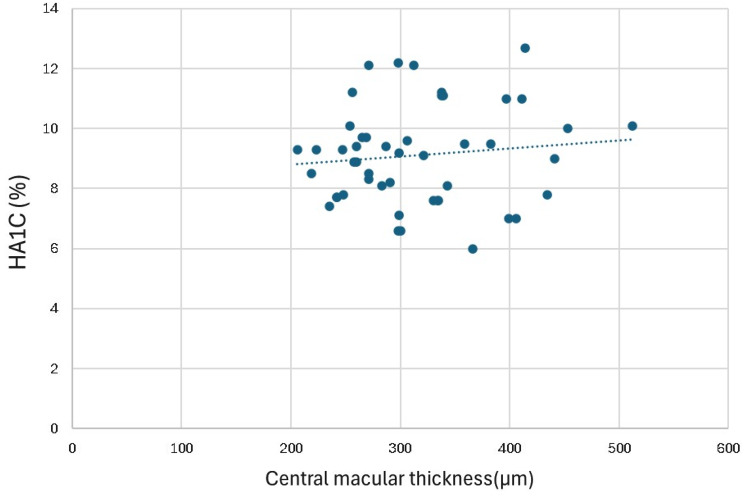
Scatter plot showing the relationship between HbA1c (%) and central macular thickness (µm) in 48 eyes diagnosed with diabetic macular edema

Among the 906 patients, 38 were diagnosed with diabetic macular edema, accounting for 4.19% of the patients. Both males and females made up 50% of the sample. The average age was 61.39 years, with a standard deviation of 10.11. A total of 31.6% of the patients had diabetes for more than 15 years. The average HbA1c level was 9.1 ± 1.7, which ranged from a minimum of 6.0 to a maximum of 12.7. In the 48 eyes with available HbA1c and OCT data, there was no significant correlation between HbA1c and central macular thickness (*r* = 0.12, *p* = 0.44) (Fig. 1).

Twenty-four patients completed the follow-up assessments. The LogMAR VA was 0.41 ± 0.53 in the right eye and 0.42 ± 0.48 in the left eye. The mean intraocular pressure (IOP) was 17.5 ± 3.2 in the right eye and 17.3 ± 2.8 in the left eye. Among the patients, 22 (91.7%) had natural lenses, while 2 (8.3%) had intraocular lenses. Additionally, 2 (8.3%) had a history of ocular trauma, 2 (8.3%) had undergone intraocular surgery, 1 (4.2%) had received Pan-retinal photocoagulation, 1 (4.2%) had undergone focal retina laser treatment, and 5 (20.8%) had been treated with intravitreal anti-VEGF therapy.

**Fig. 2:**
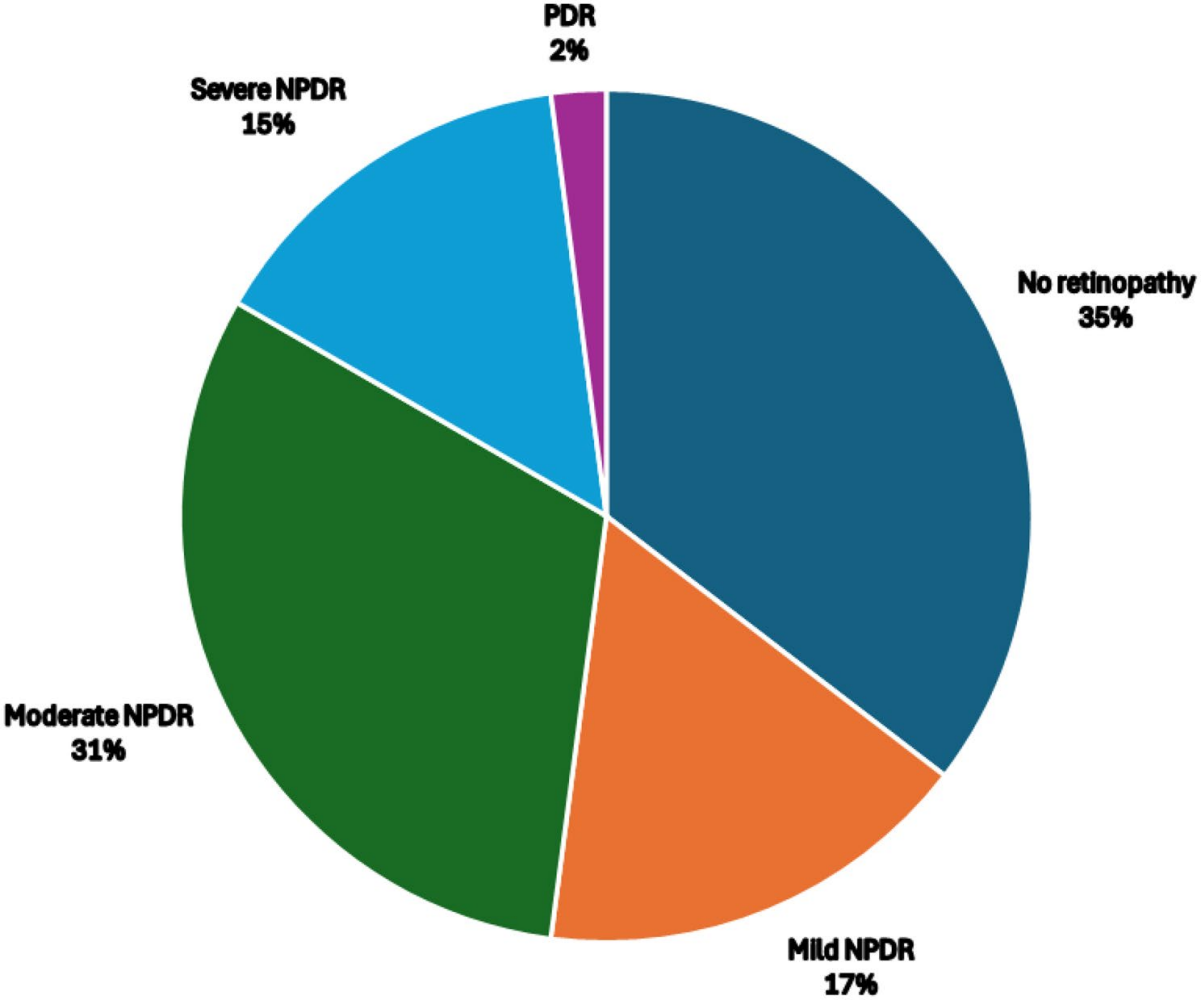
Pie chart showing the distribution of diabetic retinopathy severity in 48 eyes with diabetic macular edema. (NPDR: Non-proliferative diabetic retinopathy; PDR: proliferative diabetic retinopathy)

Forty-eight eyes were affected by diabetic macular edema, with 20 (41.7%) affected in the right eye and 28 (58.3%) in the left eye. Center-involved DME was found in 22 (45.8%) eyes, whereas non-center-involved DME was observed in 26 (54.2%) eyes. The mean central macular thickness was 315 ± 69, ranging from a minimum of 206 to a maximum of 512. Diabetic retinopathy was present in 31 eyes, representing 64.5% of the total affected eyes. Among the 48 affected eyes, 17 (35%) had no retinopathy, 8 (17%) had mild NPDR, 15 (31%) had moderate NPDR, 7 (15%) had severe NPDR, and 1 (2%) had PDR (Fig. 2).

## Discussion

This research outlines the findings from a tele-screening initiative conducted by the Ministry of National Guard Health Affairs in Riyadh, Saudi Arabia, aimed at identifying and assessing the severity of diabetic macular edema (DME). Compared with our prevalence of 4.19%, a similar study conducted in Toronto presented the results of their tele-screening program and showed similar results, with an incidence of 4.6% [11]. Additionally, they reported that 38% of those diagnosed with DME were not detected by color fundus photography alone, stressing the use of adjacent optical coherence tomography. Earlier studies have indicated that both slit-lamp bio-microscopy and stereo fundus photography lack sensitivity in detecting mild and early changes in retinal thickness [12]. On the other hand, OCT offers high-resolution retinal imaging and enables precise measurement of changes in retinal thickness or edema. Additionally, it can reveal areas of subclinical macular edema and assist in confirming the absence of macular thickening [13].

Using the Saudi National Diabetes Registry, Al-Rubeaan et al. reported that the prevalence was 5.7%, which is similar to our result, even though their study was hospital-based, and given that macular edema is a symptomatic condition, most patients were forced to seek medical help. Our 906 diabetic patients participated in a routine tele-screening visit, and a similar percentage of patients were still included [5]. In a Sweden registry, they reported a 2.5% prevalence, which could be explained by the fact that it was a population-based registry [14].

In a case‒control study published by García-Ulloa and colleagues, they compared those who were diagnosed with diabetic retinopathy and without macular edema to those who were diagnosed with diabetic retinopathy and macular edema, and in their analysis, they reported that age was a risk factor for DME [15]. Our study revealed that 86.8% of our population was older than 45 years, and one of the major risk factors for diabetic retinopathy complications is the duration of diabetes. In our cohort, hypertension and dyslipidemia were risk factors for DME, affecting most patients. The association between blood pressure and macular edema occurrence varies, with inconsistent results in both prevalence and incidence [16–18]. As stated by Aroca PR et al., elevated diastolic blood pressure can lead to increased perfusion pressure within retinal vessels, which, already compromised by hyperglycemia, may result in increased fluid leakage [17]. Moreover, the elevated average HbA1c and duration of diabetes further implicate poor glycemic control as a key contributor to DME. In our study, no significant correlation was observed between HbA1c and central macular thickness (*r* = 0.12, *p* = 0.44). This finding may be explained by the early detection of patients through the tele-screening program, which likely identified cases before significant macular thickening had developed. This is further supported by a cohort study in the UK by Martín-Merino et al. [19]. In their study, they reported that diabetic macular edema is correlated with several factors, including elevated HbA1c levels, increased systolic blood pressure, and increased total cholesterol and LDL concentrations. Comparatively, neuropathy and nephropathy, although they affect fewer patients, still signify potential microvascular compromise, which could predispose patients to retinal pathology. Suzuki Y et al. studied the relationship between DME and diabetic nephropathy and reported that DME is significantly correlated with the eGFR and albuminuria, which are indicators of diabetic nephropathy [20]. Moreover, Acan D et al. reported that 28 (44.4%) of their population had nephropathy and that 42 (66.6%) had neuropathy, indicating a relationship with DME [21]. However, in our study, only 21% of patients were diagnosed with nephropathy, and 23% were diagnosed with neuropathy, which could be due to early screening and earlier detection of diabetic retinopathy in asymptomatic patients.

In our patients, 10% were smokers. An analysis of a 25-year dataset from the Wisconsin Epidemiologic Study of Diabetic Retinopathy (WESDR) suggested an initial correlation showing increased occurrence of macular edema among diabetic patients who smoked more than 15 pack-years following their diagnosis. However, when multiple variables were considered, this correlation did not remain statistically significant [16]. In our study, the mean central macular thickness was 315 ± 69, ranging from a minimum of 206 to a maximum of 512, with 45.8% of the patients’ eyes affected by center-involved DME. This finding aligns with the observations of Saxena S et al., who suggested that targeted screening of diabetic macular edema with OCT could serve as an effective tool for assessing the severity of DME. They noted that increased central subfield thickness (CST) on SD-OCT indicates heightened VEGF activity in the retina, which suggests increased VEGF levels and correlates with greater disease severity [22]. In another study conducted by Sareddy C et al., it was recommended that all patients diagnosed with mild NPDR undergo baseline OCT, regardless of the absence of clinical signs indicating DME. This recommendation stems from their findings that increased central macular thickness (CMT) was detected in mild NPDR patients via OCT, even without any clinical evidence of macular edema [23]. Interestingly, 35% of DME-affected eyes in our cohort did not exhibit clinically detectable diabetic retinopathy on fundus photography. Possible explanations include very mild DR not captured by fundus photography, early vascular leakage detectable on OCT preceding overt retinopathy, or prior treatment that may have masked visible DR features. This observation underscores the added value of OCT in tele-screening programs, as it can identify DME in eyes that appear normal on fundus photos.

The average LogMAR VA was 0.41 ± 0.53 in the right eye and 0.42 ± 0.48 in the left eye, indicating that significant vision impairment affects patient quality of life. Rangaraju et al. reported that the mean LogMAR VA was 0.14 ± 0.16 in DME patients, which was worse than that in those without DME. This finding underscores the correlation between increased edema and decreased VA, highlighting the utility of monitoring macular and retinal thickness for tracking DME development, progression, and treatment efficacy [24]. In a systematic review conducted by Kashim RM et al., the range of sight-threatening retinopathy identified after screening varied from 11.5% to 60%. Poor compliance and repeated nonattendance are associated with an elevated risk of poor visual outcomes and an increased likelihood of developing sight-threatening retinopathy [25].

This study has some limitations. First, it was conducted in a single tertiary hospital’s tele-screening program in Riyadh, which may not represent the broader Saudi diabetic population, particularly those in rural or underserved areas. Second, the relatively short study duration (5 months) limited the ability to assess seasonal or long-term trends in DME prevalence. Third, only 24 of the 38 patients with DME completed follow-up, which affects the outcome analysis and highlights real-world barriers such as clinic accessibility and patient compliance. Finally, the relatively small number of DME cases limited our ability to perform robust multivariate regression, and risk factor associations remain descriptive. Future multi-center studies with larger cohorts and longer follow-up are warranted to improve generalizability, capture temporal variations, and identify independent risk factors.

## Conclusion

This study highlights the burden of diabetic macular edema (DME) among diabetic patients in Riyadh, Saudi Arabia, with a prevalence of 4.19%. These findings underscore the importance of regular screening for DME, especially in patients with longstanding diabetes and comorbidities such as hypertension and dyslipidemia. Early detection and intervention are important for saving vision and improving patient outcomes. Healthcare providers should prioritize diabetic eye examinations, including OCT and fundus photography, to identify and manage DME promptly. Comprehensive diabetic care strategies must be implemented to address the systemic factors contributing to diabetic complications and optimize vision preservation in this population.

## Data Availability

The datasets used and/or analyzed during the current study are available from the corresponding author upon reasonable request.

## Abbreviations

DM: Diabetes mellitus
DR: Diabetic retinopathy
NPDR: Non–proliferative diabetic retinopathy
PDR: Proliferative diabetic retinopathy
DME: Diabetic Macular Edema
OCT: Optical coherence tomography
SD: Standard deviation
µm: Micrometer
IRB: Institutional Review Board
KAIMRC: King Abdullah International Medical Research Center
EHR/EMR: Electronic health record/Electronic medical record
HbA1c/HBA1C/HA1C: Hemoglobin A1c (glycated hemoglobin)
GLP: 1–Glucagon–like peptide–1
IOP: Intraocular pressure
VA: Visual acuity
LogMAR: Logarithm of the minimum angle of resolution
VEGF: Vascular Endothelial Growth Factor
CST: Central subfield thickness
CMT: Central Macular Thickness
ICO: International Council of Ophthalmology
SNDR: Saudi National Diabetes Registry
LDL: Low–density lipoprotein
eGFR: Estimated Glomerular Filtration Rate
WESDR: Wisconsin Epidemiologic Study of Diabetic Retinopathy
SPSS: Statistical Package for the Social Sciences
